# Differential Alphavirus Defective RNA Diversity between Intracellular and Extracellular Compartments Is Driven by Subgenomic Recombination Events

**DOI:** 10.1128/mBio.00731-20

**Published:** 2020-08-18

**Authors:** R. M. Langsjoen, A. E. Muruato, S. R. Kunkel, E. Jaworski, A. Routh

**Affiliations:** a Department of Biochemistry and Molecular Biology, University of Texas Medical Branch, Galveston, Texas, USA; b Department of Microbiology and Immunology, University of Texas Medical Branch, Galveston, Texas, USA; c Sealy Center for Structural Biology and Molecular Biophysics, University of Texas Medical Branch, Galveston, Texas, USA; Indiana University Bloomington; Johns Hopkins Bloomberg School of Public Health

**Keywords:** alphavirus, chikungunya, defective RNA, viral recombination

## Abstract

Alphaviruses are positive-sense RNA arboviruses that can cause either a chronic arthritis or a potentially lethal encephalitis. Like other RNA viruses, alphaviruses produce truncated, defective viral RNAs featuring large deletions during replication. These defective RNAs (D-RNAs) have primarily been isolated from virions after high-multiplicity-of-infection passaging. Here, we aimed to characterize both intracellular and packaged viral D-RNA populations during early-passage infections under the hypothesis that D-RNAs arise *de novo* intracellularly that may not be packaged and thus have remained undetected. To this end, we generated next-generation sequencing libraries using RNA derived from passage 1 (P1) stock chikungunya virus (CHIKV) 181/clone 25, intracellular virus, and P2 virions and analyzed samples for D-RNA expression, followed by diversity and differential expression analyses. We found that the diversity of D-RNA species is significantly higher for intracellular D-RNA populations than P2 virions and that specific populations of D-RNAs are differentially expressed between intracellular and extracellular compartments. Importantly, these trends were likewise observed in a murine model of CHIKV AF15561 infection, as well as *in vitro* studies using related Mayaro, Sindbis, and Aura viruses. Additionally, we identified a novel subtype of subgenomic D-RNA that is conserved across arthritogenic alphaviruses. D-RNAs specific to intracellular populations were defined by recombination events specifically in the subgenomic region, which were confirmed by direct RNA nanopore sequencing of intracellular CHIKV RNAs. Together, these studies show that only a portion of D-RNAs generated intracellularly are packaged and D-RNAs readily arise *de novo* in the absence of transmitted template.

## Abstract (precis)

Our understanding of viral defective RNAs (D-RNAs), or truncated viral genomes, comes largely from passaging studies in tissue culture under artificial conditions and/or packaged viral RNAs. Here, we show that specific populations of alphavirus D-RNAs arise *de novo* and that they are not packaged into virions, thus imposing a transmission bottleneck and impeding their prior detection. This raises important questions about the roles of D-RNAs, both in nature and in tissue culture, during viral infection and whether their influence is constrained by packaging requirements. Further, during the course of these studies, we found a novel type of alphavirus D-RNA that is enriched intracellularly; dubbed subgenomic D-RNAs (sgD-RNAs), they are defined by deletion boundaries between the capsid-E3 region and the E1-3′ untranslated region (UTR) and are common to chikungunya, Mayaro, Sindbis, and Aura viruses. These sgD-RNAs are enriched intracellularly and do not appear to be selectively packaged, and additionally, they may exist as subgenome-derived transcripts.

## INTRODUCTION

The genus *Alphavirus* encompasses many important human pathogens, including the equine encephalitis viruses, chikungunya virus (CHIKV), Sindbis virus (SINV), and Mayaro virus (MAYV), among others. Pathogenic alphaviruses are transmitted by a mosquito vector and generally follow the same replication strategy despite infecting distinct organisms and tissues. The alphavirus genome consists of single-stranded positive-sense RNA and carries 4 nonstructural genes, nsP1 to nsP4, and 5 structural genes, encoding capsid, E3, E2, TF/6K, and E1. The viral genome is capped and polyadenylated, allowing translation of the nonstructural genes upon entry of viral genomic RNA in the cytosol. From this, the viral replication machinery synthesizes antisense genomes, which are subsequently used to synthesize new genomic sense RNA (1). A key feature of the alphavirus genome is a subgenomic (sg) promoter, which controls expression of sgRNA from antisense template RNA independently of its genomic-length counterpart. This sgRNA encodes the structural polyprotein, which includes all the structural proteins.

Viral RNA polymerases are notoriously error prone and with few exceptions lack error-correcting capabilities. While this is most often associated with high mutation rates, these errors frequently include recombination events that cause deletion and/or duplication events (2). This can result in truncated and nonviable genomes that are termed defective RNAs (D-RNAs) or defective viral genomes (DVGs). Here, we use the term “D-RNA,” as this is descriptively broader and better suits the results of this particular work, although both terms have been used in both historical and contemporary publications. D-RNAs are replication defective alone but can be replicated, packaged, and propagated in the presence of wild-type (WT) or helper virus provided that they retain the minimally required functional motifs in their genomes (3). Our current understanding of D-RNAs comes largely from *in vitro* passaging studies that used a high multiplicity of infection (MOI, or infectious units per cell) or undiluted virus over the course of multiple viral passages. In this way, D-RNAs have been discovered for nearly every pathogenic RNA virus family in tissue culture (4–12) and are referred to as defective interfering RNAs (or particles) when they have the demonstrated ability to attenuate wild-type virus replication in tissue culture or in animal models of disease. Additionally, they have been proposed to promote viral persistence (13, 14). Although D-RNAs have been considered an epiphenomenon of cell-culturing practices, emerging deep-sequencing technologies have enabled researchers to uncover D-RNAs in both natural and laboratory animal infections (15, 16), as well as in human samples (17, 18).

Through sequencing of *in vivo* D-RNA populations, it is now emerging that D-RNAs play important roles in the outcome of disease in a number of studies. For example, respiratory syncytial virus (RSV) D-RNAs isolated from human nasopharyngeal samples were correlated with heightened immune response to infection and subsequently with improved patient outcomes (17). Similarly, D-RNAs have been isolated from both animals and humans infected with various influenza A virus (IAV) strains (18–21), many of which are similar to those observed *in vitro* (15). Interestingly, a mutation in the viral RNA polymerase that reduced production and subsequent accumulation of D-RNAs was associated with increased influenza disease severity in both humans and mice (18); further, virus isolates from humans with severe IAV infection outcomes contained smaller amounts of D-RNAs than those from mild outcomes. Like RSV D-RNAs, IAV D-RNAs are thought to be immunostimulatory and therefore lead to improved patient outcomes (18, 22–24). However, it has also been proposed that large amounts of D-RNAs present in a live-attenuated vaccine formulation can lead to decreased vaccination efficacy by suppressing vaccine strain replication and subsequent immune stimulation (21). Thus, biological functions of D-RNAs, as well as their potential use in vaccines and therapeutics, are of increasing interest.

Alphavirus D-RNAs were first formally described for Semliki Forest virus (SFV) (25, 26) and SINV (27), two prototypical Old World alphaviruses. As with other viral D-RNAs, these alphavirus D-RNAs were generated by undiluted or high-MOI serial passaging and were found to suppress wild-type parental virus replication. Later, SINV D-RNA production in persistently infected BHK cells was positively correlated with increased resistance to SFV challenge (28), indicating a potential for D-RNAs to interfere with the replication cycles of heterologous alphaviruses. The first alphavirus D-RNAs to be sequenced were derived from passage 11 (P11) SFV; Lehtovaara et al. subsequently found that SFV D-RNAs contained conserved nucleotide sequences from the most 5′ and 3′ ends of the genome, which were rearranged across several repeats (29, 30). Similar deletions spanning the majority of the two open reading frames were later identified for SINV (31), although Monroe et al. additionally found that D-RNA populations were generally heterogeneous and consisted of numerous species. From these and other studies (25–27, 32, 33), it has been hypothesized that alphavirus D-RNAs are encoded by defective negative-sense templates that are subsequently transcribed into defective positive-sense transcripts. This hypothesis is supported by the discovery of novel double-stranded intracellular RNA species in late-passage SINV infections (32), which suggests the presence of a truncated intermediate. Later, a particularly common deletion spanning from the nsP1 to the E1 genes of the SINV genome was described in the context of a low-fidelity SINV polymerase mutant (34). More recently, CHIKV has been shown to generate recombinant RNAs in tissue culture, especially RNAs featuring complex duplication and deletion events in the 3′ untranslated region (UTR) (34–39). While D-RNAs have not yet been described for CHIKV in either natural or laboratory animal infections, D-RNAs generated *in vivo* have been identified for other alphaviruses. For example, D-RNAs have been found for the distantly related salmonid alphavirus 3 in both salmon from Atlantic farms as well as experimentally infected salmon (16, 40). Additionally, 6K deletion mutants were identified in a Venezuelan equine encephalitis virus (VEEV) isolate from a sentinel hamster (41). Finally, SINV D-RNAs have been recovered from experimentally infected *Drosophila* (39).

Despite increasing interest in the biological consequences of D-RNA production, the majority of D-RNA research still generally relies on identifying D-RNAs through serial *in vitro* passaging studies. Moreover, all D-RNA studies to date have focused on packaged D-RNA populations, assaying intracellular compartments only after D-RNAs have accumulated over multiple passages. Thus, important questions about the biogenesis and intracellular functions of D-RNAs produced *de novo* remain largely unexplored. In this study, we characterized both intracellular and packaged D-RNA populations during early viral passages, under the hypothesis that numerous D-RNAs arise *de novo* intracellularly that are not packaged. By thoroughly investigating intracellular RNA diversity and its relation to packaged RNA populations, we can define D-RNA subtypes and thereby elucidate specific roles D-RNAs play during infection. To this end, we used Illumina sequencing to sequence RNA from P1 alphavirus stock, intracellular RNA (P1.5), and resultant P2 virion RNA, and we used various bioinformatics approaches to analyze differences in D-RNA diversity and expression. Intracellular D-RNA expression was also evaluated using Oxford Nanopore Technologies’ direct RNA sequencing. Finally, we utilized a murine model of CHIKV infection to demonstrate that these *in vitro* trends hold true in a complex biological system.

## RESULTS

### Intracellular and virion CHIKV D-RNA populations are distinct.

To investigate potential differences between intracellular and packaged D-RNA populations during early passages, African green monkey kidney cells (Vero) were infected with CHIKV 181/clone 25 at a multiplicity of infection (MOI) of 2. After 12 h of incubation, cells were washed three times with phosphate-buffered saline (PBS), and then total cellular RNA was extracted and rRNA depleted. Concomitant infections were incubated for 48 h, and then supernatant was collected, clarified by centrifugation, and concentrated using polyethylene glycol (PEG)-NaCl precipitation; concentrated virus was incubated with 2 μg RNase A for 1 h at room temperature to remove nonpackaged RNAs prior to RNA extraction. By following this study design, we were able to maximize available viral RNA from both compartments and thus identify rare events. RNA sequencing (RNA-seq) libraries were then constructed using the previously described ClickSeq library preparation method optimized for the discovery of rare recombination events (42, 43) and sequenced on an Illumina NextSeq550 system. ClickSeq reduces artifactual recombination by eliminating the fragmentation step of standard Illumina library preparation and is also ideal for use with low-quality or degraded samples (42). Reads were trimmed and filtered using fastp (44) and analyzed using the ViReMa v1.5 pipeline, which provides alignment data, recombination events, and associated count data (19, 43, 45). This study design was repeated once with one replicate and a second time with three replicates (used for statistical analyses). Additionally, RNA from two separate stock vials was purified and sequenced. These were not included in statistical comparisons but are shown nonetheless to establish a baseline D-RNA phenotype. CHIKV 181/clone25 is a vaccine strain derived from serial passaging of the AF15561 Asian strain and has putative attenuating mutations in the E2 protein (46, 47). It was selected for these baseline studies due to its lower containment requirements. We do not expect the attenuating mutations of this strain to affect D-RNA production, as the attenuating mutations are in the structural genes and RNA replication is not attenuated *in vitro* (47). All reactions resulted in robust sequencing read coverage across the reference virus genome, with median nucleotide coverage ranging between 34,268 and 71,485 reads (Table 1). Median coverage was slightly higher for nucleotides in the subgenomic region in intracellular samples, ranging between 5.4 and 5.9 times higher than those in the nonstructural gene cassette. In general, Us were enriched immediately upstream of donor junctions, while Cs were observed less frequently at this same position, with a similar trend in acceptor junctions (Fig. S1).

**TABLE 1:** Sequencing reaction data for chikungunya virus 181/clone25 ClickSeq libraries^*a*^

Reaction	No. of reads			Median nt coverage
	Total	Host	Virus	
Stock A	14,518,846	199,561	13,750,288	109,863.5
Stock B	12,915,787	212,293	12,191,588	100,215
IC1	12,097,983	2,074,592	9,269,428	36,789
IC2	9,180,835	1,581,780	6,995,805	29,397
IC3	10,342,573	1,784,129	7,903,445	34,267.5
V4	10,572,563	132,470	9,773,653	71,485
V5	8,796,035	153,536	8,063,893	56,349
V6	6,792,885	171,694	6,209,907	45,663

a IC, intracellular; V, virion; nt, nucleotide.

10.1128/mBio.00731-20.1 FIG S1 Distribution of nucleotides at CHIKV recombination junctions. Three replicates of intracellular RNA from chikungunya virus (CHIKV)-infected Vero cells were collected 12 h post-infection and then sequenced and evaluated for defective RNA expression using ViReMa v1.5. Nucleotides observed upstream and downstream of recombination junctions were then enumerated and converted to percentage of recombination events. Native nucleotide distribution (expected) is indicated by solid black lines, intracellular samples are indicated by blue lines, and P2 virion samples are indicated by pink lines. (Top) donor sites with nucleotides A, U, G, and C (left to right); (bottom) acceptor sites with nucleotides A, U, G, and C (left to right). Download FIG S1, TIF file, 0.8 MB. Copyright © 2020 Langsjoen et al. 2020 Langsjoen et al. This content is distributed under the terms of the Creative Commons Attribution 4.0 International license.

**FIG 1:**
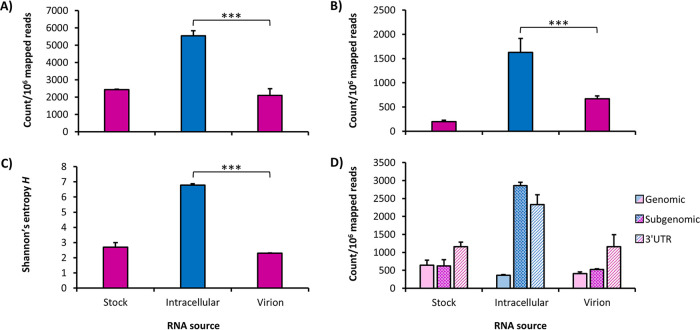
Overview of D-RNA expression in P1 stock, intracellular, and P2 RNA samples. RNA from two replicates of passage 1 (P1) stock virus, three replicates of intracellular virus (P1.5), and three replicates of resulting P2 virion RNA from CHIKV-infected Vero cells and supernatant were sequenced and analyzed for D-RNA expression using ViReMa v1.5. D-RNA count data were then normalized to count per 10^6^ reads, and the total number of D-RNAs/10^6^ mapped reads (A) and the unique D-RNAs/10^6^ mapped reads (B) were calculated for each compartment. This was followed by calculating Shannon’s diversity index (*H*) (C), weighted for median nucleotide coverage, using a custom python script. Pink indicates samples taken from virions (P1 stock and P2); blue indicates intracellular samples. ***, *P* < 0.001 (Student’s *t* test). (D) The types of total recombination events were further broken down into genomic (those with donor sites in the nonstructural coding region), subgenomic (those with donor sites in the structural coding region), and 3′ UTR (those with donor and acceptor sites within the 3′ UTR) for each RNA source.

Overall, after normalizing count data to the number of mapped reads per million sequenced reads, both the total number of recombination events and the number of unique events were significantly higher among intracellular RNAs than virion RNAs (Student’s *t* test, *P* < 0.01 for all) (Fig. 1A and B), indicating that the majority of D-RNAs generated during replication were not packaged. This is supported by Shannon’s entropy analysis, calculated from raw D-RNA count data weighted against median wild-type coverage as previously described (43), which found that Shannon’s diversity index (*H*) for D-RNAs is significantly higher for intracellular RNAs than virion-derived RNAs (Student’s *t* test, *P* ≪ 0.005) (Fig. 1C); thus, intracellular CHIKV D-RNA populations are more diverse than P2 virions, indicating a bottleneck imposed by the packaging process. The distribution of recombination event types (i.e., those with donor sites in the nonstructural or genomic coding region, in the structural or subgenomic coding region, and within the 3′ UTR) also differs between intracellular and extracellular compartments, with 3′ UTR events generally enriched in P2 virions and subgenomic events highly enriched in the intracellular compartment (Fig. 1D).

**FIG 2:**
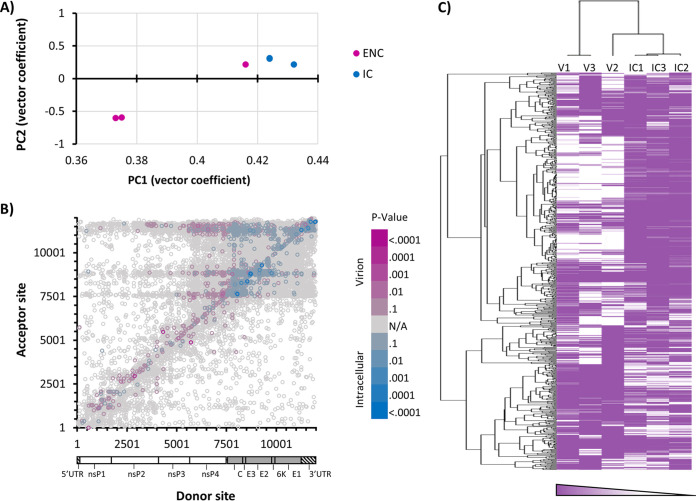
Characterization of CHIKV D-RNA populations using differential expression approaches. Three replicates each of intracellular (blue) and resulting virion (pink) RNA from CHIKV infected Vero cells and supernatant, respectively, were sequenced and evaluated for defective RNA (D-RNA) expression using ViReMa v1.5. Count tables of D-RNAs abundance were passed to DESeq2 for subsequent analyses. (A) Two-dimensional principal-component analysis; (B) differential expression of all D-RNA species; (C) hierarchical clustering of CHIKV D-RNA species expression (using normalized count number from DESeq2 output with Cluster 3.0 and Treeview).

A principal-component analysis (PCA) of recombination event count frequencies was performed for intracellular and P2 virion D-RNAs (Fig. 2A), which revealed that intracellular samples cluster together, while two of the three P2 virion replicates cluster together, with the third clustering being closer to intracellular samples, indicating that variation in D-RNA expression patterns is mostly unique to its respective sources (intracellular versus P2 virion). This is supported by differential expression analysis (Fig. 2B and C), performed with DESeq2 (48) followed by hierarchical clustering with Cluster 3.0 (49) and TreeView (50), which revealed that D-RNAs with recombination events found in distinct genomic regions are differentially enriched in each RNA source. Of note, recombination events enriched intracellularly occur within the subgenomic region. Altogether, these data demonstrate that intracellular and P2 virion D-RNA populations differ significantly by all metrics employed, confirming that many D-RNAs arise *de novo* that are not efficiently packaged.

### Subgenomic deletions: a new D-RNA archetype for CHIKV.

**FIG 3:**
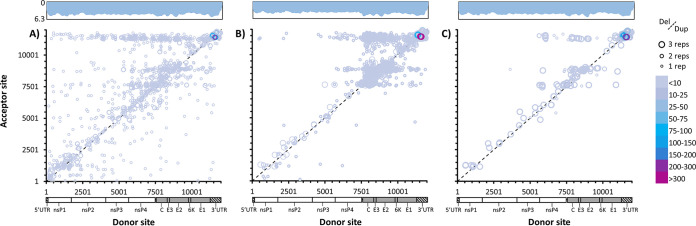
Recombination boundaries observed among CHIKV D-RNAs. CHIKV recombination junctions for D-RNAs with ≥1 count per every 2 × 10^6^ reads. RNA was derived from P1 stock virus (two replicates) (A), intracellular RNA (three replicates) from infected Vero cells (B), and resulting P2 virions (three replicates) (C). Circle size indicates number of replications, and lines delineate deletion versus duplication-insertion events, while color indicates count/10^6^ mapped reads. Log coverage data are shown above the graphs, with light blue indicating average coverage over three replicates and dark blue the standard deviation.

**FIG 4:**
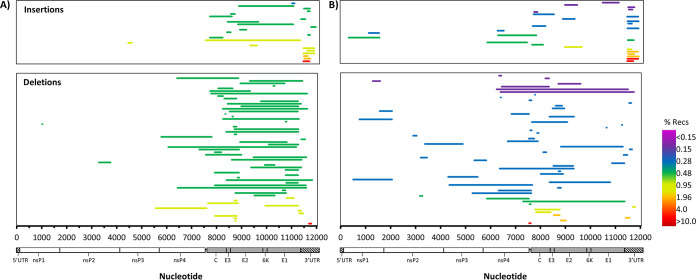
Top recombination events observed among CHIKV 181/clone25 D-RNAs. One replicate of each of intracellular RNA from CHIKV-infected Vero cells collected 12 h post-infection (hpi) (A) and supernatant collected 48 hpi (B) was sequenced and evaluated for defective RNA (D-RNA) expression using ViReMa v1.5. Recombinant count data were normalized to count per 10^6^ CHIKV mapped reads, and the top 80 recombination events were split into insertion-duplication events (top) and deletion events (bottom). Color represents the percentage of overall recombination events represented by a single event.

To compare specific boundaries, all normalized ViReMa count data from each data set (stock, intracellular, and virion) were combined; normalized count data for D-RNAs present in at least two of three samples were averaged, and then the boundaries for D-RNAs with at least 1 count per 2 × 10^6^ mapped reads were illustrated using heat maps, along with associated coverage data (Fig. 3). While several distinct and abundant acceptor sites (indicated by horizontal striations) appear in all three data sets around nucleotides 7750, 8850, and 11500, subgenomic recombination events are enriched among intracellular RNAs compared to both stock and virion samples (specific nucleotides frequencies are shown in Fig. S2 in the supplemental material). To further visualize this, the top 80 recombination events for intracellular and virion RNAs were divided into “duplication” or “back-splicing” (“insertion”) events and deletion events and then mapped (Fig. 4; one representative replicate is shown). Several major D-RNA archetypes emerge: deletion events between the nsP4 region and the end of the E1-3′ UTR regions, which are common among both intracellular and virion RNAs; deletion events between the capsid-E3 region and the E1-3′ UTR region, which are common intracellularly but are relatively rare among virion RNAs; and deletion and duplication events occurring specifically within the 3′ UTR, which are among the most common in both intracellular and virion RNAs (subgenomic recombination types among intracellular RNAs are illustrated in Fig. S3). This illustrates that the compositions of intracellular and virion D-RNA populations differ substantially, while also demonstrating novel D-RNA archetypes not previously observed in alphaviruses.

10.1128/mBio.00731-20.2 FIG S2 Recombination junction counts for CHIKV D-RNAs. Three replicates of intracellular RNA from chikungunya virus (CHIKV)-infected Vero cells were collected 12 h post-infection and then sequenced and evaluated for defective RNA expression using ViReMa v1.5. Recombinant count data were normalized to count per 10^6^ CHIKV-mapped reads, and then average nucleotide count for donor sites (top) and acceptor sites (bottom) were plotted. Download FIG S2, TIF file, 0.5 MB. Copyright © 2020 Langsjoen et al. 2020 Langsjoen et al. This content is distributed under the terms of the Creative Commons Attribution 4.0 International license.

10.1128/mBio.00731-20.3 FIG S3 CHIKV subgenomic recombination junctions. Three replicates of intracellular RNA from chikungunya virus (CHIKV)-infected Vero cells were collected 12 h post-infection and then sequenced and evaluated for defective RNA expression using ViReMa v1.5. Recombinant count data were normalized to count per 10^6^ CHIKV-mapped reads, and then subgenomic recombination events (both junctions [Jct], occurring after nucleotide 7500) were averaged and then ranked from highest to lowest count; the top 200 in total are shown. Top line, donor site (Jct 1); bottom line, acceptor (Jct 2). (A) 3′ UTR-specific recombination events; (B) subgenomic deletions; (C) subgenomic insertion/duplication events. Download FIG S3, TIF file, 1.0 MB. Copyright © 2020 Langsjoen et al. 2020 Langsjoen et al. This content is distributed under the terms of the Creative Commons Attribution 4.0 International license.

### Direct RNA sequencing of CHIKV RNA differentiates subgenomic D-RNAs and genomic D-RNAs.

For the recombination events observed in the subgenomic region in intracellular samples, it is possible that they occur on either genomic or sg transcripts. Thus, to put these subgenomic deletions (sgDels) into context, two separate Oxford Nanopore Technologies (ONT) direct RNA sequencing (DRS) libraries were generated using two intracellular samples, extracted as described above at 12 h post-infection (hpi) (derived from independent infections that utilized completely distinct CHIKV 181/clone 25 stocks). ONT DRS directly sequences the input RNA on a single-molecule-by-molecule basis (there is no PCR or amplification process), maintaining native features of the RNA (51). Therefore, this technology can robustly quantify relative numbers of genomic and sgRNA reads. Reads were base-called using Albacore v2.0.1 and aligned to the CHIKV 181/clone 25 genome using minimap2 (52). Full-length reads were recovered (totaling 75 to 82 reads per data set), and median nucleotide coverage ranged from 229 to 249.5, with decreasing coverage from the 3′ UTR [due to 3′-to-5′ sequencing from the poly(A) tail] and a sharp drop in coverage following the 5′ UTR of the sgRNA (Fig. S4), showing a clear difference between genomic and subgenomic reads. From aligned data, specific read junctions were extracted from the SAM file, and reads containing deletions were assessed (Table 2). For both sequencing data sets, multiple deletion events on a single read were rare, with 96 to 98% of deleted reads featuring just one deletion.

**TABLE 2:** Sequencing reaction data for chikungunya virus 181/clone25 direct RNA sequencing libraries^*a*^

Reaction	No. of reads		Median nt coverage	No. of deletions			
	Total	Mapped		Single	Genomic	sg	Unclass
DRS1	60,233	37,509	249.5	1,077	212	216	705
DRS2	72,457	19,791	229	864	142	200	522

a nt, nucleotide; unclass, unclassified.

10.1128/mBio.00731-20.4 FIG S4 Coverage data for CHIKV MinION direct RNA sequencing reactions. Two replicates of intracellular RNA from chikungunya virus (CHIKV)-infected Vero cells were collected 12 h post-infection, and then two separate sequencing libraries were constructed using Oxford Nanopore Technologies’ direct RNA sequencing kit and sequenced on a MinION sequencer. After aligning reads to the CHIKV genome, log_10_ nucleotide coverage for both reactions (DRS1 [A] and DRS2 [B]) was plotted. Download FIG S4, TIF file, 0.4 MB. Copyright © 2020 Langsjoen et al. 2020 Langsjoen et al. This content is distributed under the terms of the Creative Commons Attribution 4.0 International license.

**FIG 5:**
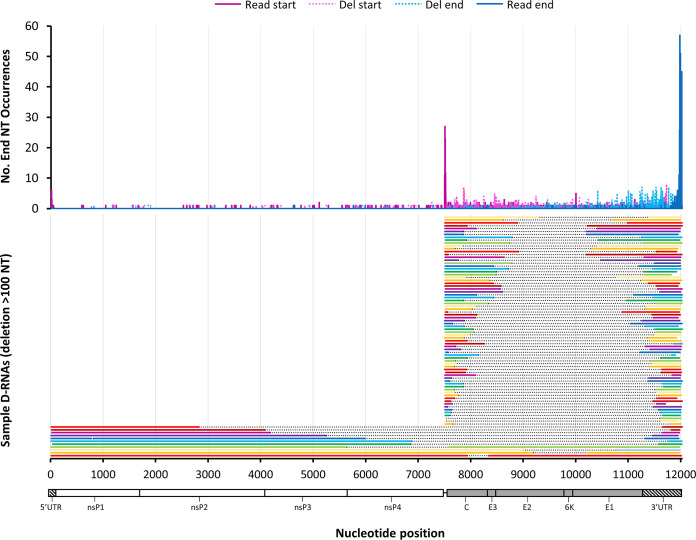
Oxford Nanopore’s direct RNA sequencing of intracellular CHIKV D-RNA species. Intracellular RNA from CHIKV-infected Vero cells was sequenced using Oxford Nanopore’s direct RNA sequencing kit on a MinION sequencer, reads were mapped using minimap2, and then read boundaries for CHIKV-mapped reads were extracted from SAM files. Boundary counts for defective RNA reads containing deletions were enumerated and plotted (top), and the raw single reads containing the largest deletions for genomic and subgenomic D-RNAs are shown (bottom).

First, reads were placed into one of two categories: (i) genomic reads containing deletions, defined as reads beginning between nucleotides (nt) 1 and 7450, and (ii) sg reads containing deletions, defined as reads beginning between nt 7500 and 7550. Beyond these nucleotides, it is not possible to discern between true sgRNA reads and genomic reads that may have simply been truncated during the sequencing process (either due to RNA fragmentation or incomplete 3′-to-5′ nanopore sequencing of the RNA). The largest deletions for genomic and sgRNA reads are shown for one of the libraries (Fig. 5; one representative replicate is shown), along with read and deletion boundary counts. These data confirm that many of the sgDels occur specifically on sgRNAs rather than genomic RNAs. Interestingly, although sgDels were present in both genomic and sgRNA transcripts, there was little or no commonality between sgDels observed in genomic reads and sgDels observed in sg reads, with only two sgDels being common to both sgRNA and genomic RNA reads in DRS1. Thus, we were unable to confirm the presence of a common defective template for the overwhelming majority of sgDels observed.

Finally, these data additionally reveal that 2.9 to 3.2% of all aligned reads from intracellular samples contained at least one deletion, calculated by dividing mapped reads with deletions by total mapped reads. Using the same calculation with Illumina data, the overall percentage of reads containing any recombination event from intracellular samples ranged from 0.2642 to 0.2842%. This difference is likely due to reverse transcription- and PCR-based biases associated with the Illumina platform (53–55), which can cause variation in nucleotide coverage across the genome and thus necessitate the consideration of local nucleotide coverage when percent D-RNAs is calculated. Calculating percent D-RNAs for alphaviruses is further complicated by the molar disparity between genomic and sgRNA expression. Adjusting Illumina count percentages by average nucleotide coverage for a specific region—genomic or subgenomic—suggests that D-RNAs may make up 9.79 ± 1.25% of intracellular populations and 7.65 ± 1.26% of virion populations. However, there is no universally accepted method to calculate D-RNA proportions using short-read data, whereas proportions can be calculated from DRS data using straightforward ratios (56).

### D-RNA expression patterns are conserved across the alphavirus family.

To ascertain whether sgD-RNAs are a CHIKV-specific phenomenon or conserved broadly among arthritogenic alphaviruses, the above-described *in vitro* studies were repeated using Mayaro virus (MAYV), Sindbis virus (SINV), and Aura virus (AURV) (Table 3). Of these, MAYV shares the highest sequence identity with CHIKV at 62.91% and subsequently groups in the same Semliki Forest virus complex, while SINV and AURV group phylogenetically with the adjacent Western equine encephalitis virus (WEEV) complex (57). Additionally, AURV is the only alphavirus that has been shown to package its sgRNA to date (58–60). Similar to CHIKV, Cs were enriched immediately upstream of donor junctions, while Gs were enriched immediately upstream of AURV donor junctions and Us were enriched immediately downstream; Cs were observed less frequently surrounding donor sites for all (Fig. S5). Similar trends were observed for acceptor junctions (Fig. S6)

**TABLE 3:** Sequencing reaction data for alphavirus ClickSeq libraries^*a*^

Virus	Reaction	No. of reads			Median nt coverage
		Total	Host	Virus	
MAYV	Stock A	11,237,488	739,309	9,752,483	62,911.5
	Stock B	7,844,806	537,876	6,752,470	43,501
	IC1	7,768,828	224,809	7,193,754	29,647
	IC2	13,462,430	482,136	12,316,587	59,411.5
	IC3	13,785,735	497,972	12,650,339	60,552
	V4	15,579,050	318,665	14,372,458	99,067.5
	V5	9,744,511	51,403	9,191,490	51,931.5
	V6	12,378,399	562,539	10989209	77,123

SINV	Stock A	15,645,771	124,090	14,523,317	135,460
	Stock B	21,685,524	229,835	20,058,161	193,799
	IC1	13,147,461	629,605	11,670,451	123,197.5
	IC2	14,889,134	758,124	13,132,843	141,347.5
	IC3	24,139,976	1,213,680	21,223,018	235,621
	V4	13,502,412	142,151	12,439,978	123,633
	V5	8,357,462	97,234	7,618,128	76,468
	V6	11,169,412	152,582	10,244,173	104,177.5

AURV	Stock A	13,920,389	788,247	12,360,809	109,459
	Stock B	44,224	3,716	32,072	310
	IC1	17,124,283	4,019,278	3,052,443	23,515.5
	IC2	16,693,920	10,549,065	1,746,381	14,004
	IC3	12,922,934	7,390,912	2,596,651	21,329
	V4	13,609,695	933,635	6,935,105	70,177.5
	V5	23,500,149	3,428,681	1,063,597	9,852
	V6	9,315,406	3,537,249	3,985,563	39,441.5

a IC, intracellular; V, virion; nt, nucleotide.

10.1128/mBio.00731-20.5 FIG S5 Distribution of nucleotides at MAYV, SINV, and AURV donor sites. Three replicates of intracellular RNA from Mayaro virus (MAYV)-, Sindbis virus (SINV)-, and Aura virus (AURV)-infected Vero cells were collected 12 h post-infection and then sequenced and evaluated for defective RNA expression using ViReMa v1.5. Nucleotides observed upstream and downstream of donor junctions were then enumerated and converted to percentage of recombination events. Native nucleotide distribution (expected) is indicated by solid black lines, intracellular samples are indicated by blue lines, and P2 virion samples are indicated by pink lines. MAYV donor sites (top), SINV donor sites (middle), and AURV donor sites (bottom) with nucleotides A, U, G, and C (left to right) are shown. Download FIG S5, TIF file, 1.1 MB. Copyright © 2020 Langsjoen et al. 2020 Langsjoen et al. This content is distributed under the terms of the Creative Commons Attribution 4.0 International license.

10.1128/mBio.00731-20.6 FIG S6 Distribution of nucleotides at MAYV, SINV, and AURV donor sites. Three replicates of intracellular RNA from Mayaro virus (MAYV)-, Sindbis virus (SINV)-, and Aura virus (AURV)-infected Vero cells were collected 12 h post-infection and then sequenced and evaluated for defective RNA expression using ViReMa v1.5. Nucleotides observed upstream and downstream of donor junctions were then enumerated using and converted to percentage of recombination events. Native nucleotide distribution (expected) is indicated by solid black lines, intracellular samples are indicated by blue lines, and P2 virion samples are indicated by pink lines. MAYV acceptor sites (top), SINV acceptor sites (middle), and AURV acceptor sites (bottom) with nucleotides A, U, G, and C (left to right) are shown. Download FIG S6, TIF file, 1.1 MB. Copyright © 2020 Langsjoen et al. 2020 Langsjoen et al. This content is distributed under the terms of the Creative Commons Attribution 4.0 International license.

**FIG 6:**
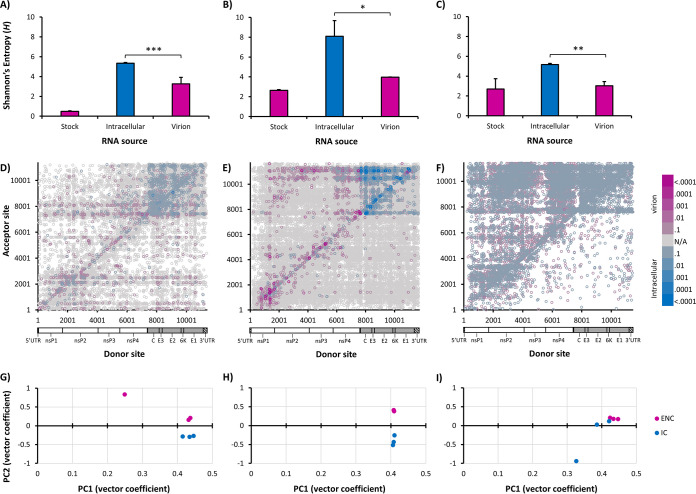
Characterization of alphavirus D-RNA populations using differential expression approaches. Three replicates each of intracellular (blue) and resulting virion (pink) RNA from Mayaro virus-infected (A, D, and G), Sindbis virus-infected (B, E, and H), and Aura virus-infected (C, F, and I) cells and supernatant was sequenced and evaluated for defective RNA (D-RNA) expression using ViReMa v1.5. DESeq2 was then used to concatenate and normalize count data for subsequent analyses. (A through C) Shannon’s diversity index *H*; (D through F) differential expression of all D-RNA species; (G through I) principal-component analyses. *, *P* < 0.05; **, *P* < 0.01; ***, *P* < 0.001.

For all three additional alphavirus species, D-RNA diversity was significantly higher intracellularly than among virion RNAs (Fig. 6A to C), again indicating both a packaging bottleneck in the transmission of D-RNAs and the potential for D-RNAs to arise *de novo* without the aid of a transmitted template. Interestingly, the two New World representatives, MAYV and AURV, shared similar intracellular and virion D-RNA diversity indices, whereas the two Old World representatives, CHIKV and SINV, shared similar intracellular, but not virion, indices. Further, these differences in population diversity, as with CHIKV, were largely driven by recombination events in the subgenomic region for MAYV (Fig. 6D). Recombination events in the SINV subgenome were similarly enriched in intracellular samples (Fig. 6E), while additional recombination events in and across the nonstructural cassette were significantly enriched in virion samples, including previously described D-RNAs (34). AURV appeared to have a bimodal distribution of differentially expressed D-RNAs, thus making interpretation of these results unreliable (Fig. 6F). PCA indicated that intracellular and virion samples clustered independently for both MAYV and SINV (Fig. 6G and H). However, while virion AURV samples clustered together, intracellular samples showed no clustering pattern (Fig. 6I).

**FIG 7:**
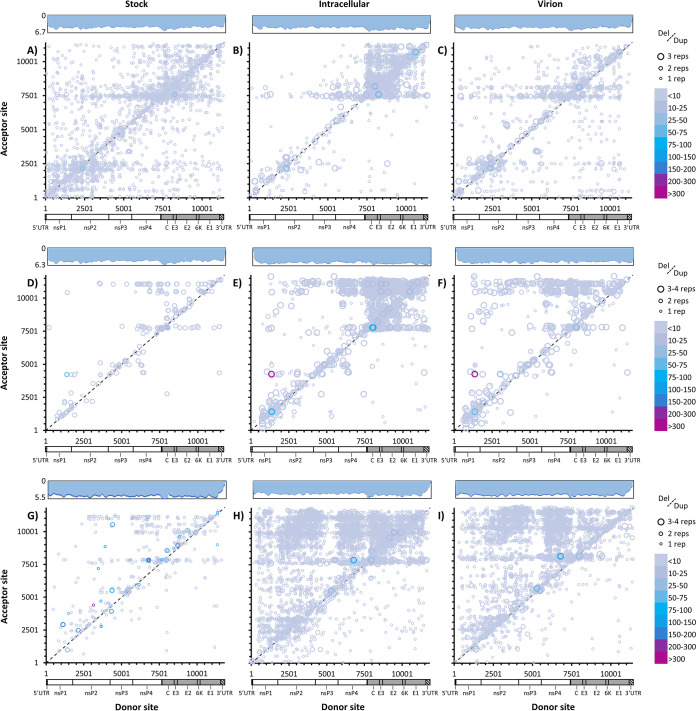
Recombination boundaries observed among alphavirus D-RNAs. Alphavirus recombination junctions for D-RNAs with ≥1 count/2 × 10^6^ reads from stock RNA (left panels), intracellular RNA (center panels), and virion RNA (right panels) for the Mayaro virus (A to C), Sindbis virus (D to F), and Aura virus (G to I) were assessed. Circle size indicates number of replications, lines delineate deletion versus duplication-copyback events, and color indicates count/10^6^ mapped reads. Log coverage data are shown above the graphs, with light blue indicating average coverage over three replicates and dark blue the standard deviation.

Furthermore, heat maps of recombination junctions reveal that each of these additional alphaviruses showed a strong acceptor site near the subgenomic promoter that carried over from stock through P2 virion RNAs (horizontal striations in Fig. 7), similar to that observed for CHIKV: for MAYV, near nt 7600 (Fig. S7A); for SINV, near nt 7760 (Fig. S7B); and for AURV, near nt 7840 (Fig. S7C). These sites were accompanied by an additional downstream donor site that was nearly absent in both stock and P2 virion RNAs, recapitulating the pattern observed for CHIKV. However, while MAYV especially exemplifies this pattern, SINV showed signs of a donor site in both stock and P2 virion samples. MAYV showed several additional acceptor sites in the nonstructural genes, as well as one acceptor site near nt 8030. Unlike all other alphaviruses, an acceptor site was observed near the end of the MAYV E1 gene in intracellular samples and only weakly in stock virus. On the other hand, SINV and AURV both displayed acceptor sites near the end of their respective E1 genes in all three sample types. AURV additionally displayed a variety of both donor and acceptor sites and heavily favored deletions over “duplications” in all sample types, aggregating in hot spots in the nsP2 and nsP4 genes.

10.1128/mBio.00731-20.7 FIG S7 Recombination junction counts for MAYV D-RNAs. Three replicates of intracellular RNA from Mayaro virus (MAYV) (A)-, Sindbis virus (SINV) (B)-, and Aura virus (AURV) (C)-infected Vero cells were collected 12 h post-infection and then sequenced and evaluated for defective RNA expression using ViReMa v1.5. Recombinant count data were normalized to count per 10^6^ virus-mapped reads, and then average nucleotide count for donor sites (top) and acceptor sites (bottom) were plotted for each. Download FIG S7, TIF file, 1.0 MB. Copyright © 2020 Langsjoen et al. 2020 Langsjoen et al. This content is distributed under the terms of the Creative Commons Attribution 4.0 International license.

### Murine infection with wild-type CHIKV results in D-RNA expression patterns similar to those in early-passage Vero cell infections.

To assess whether *in vitro* D-RNA expression patterns are recapitulated *in vivo*, alpha interferon receptor-null (IFN-αR^−/−^) (A129) mice were infected with 10^3^ PFU of CHIKV AF15561, the parental strain to CHIKV 181/clone 25 (46, 61), and monitored for 4 days post-infection (dpi) until clinical signs such as ruffled fur and hunched posture appeared; serum, skeletal muscle from injected and contralateral limbs, heart, kidney, liver, and spleen were collected upon humane euthanization at 4 dpi. Virus in serum was quantified by plaque assay, while RNA was extracted from all samples using either TRIzol or a Qiagen RNeasy fibrous tissue kit. rRNA was depleted from tissue samples, and then ClickSeq libraries were synthesized for next-generation sequencing (NGS). Fewer than 10,000 reads from most liver and spleen samples mapped to the CHIKV genome despite robust sequencing reactions (Table 4); therefore, spleen and liver were excluded from downstream analyses.

**TABLE 4:** Sequencing reaction data for ClickSeq libraries from chikungunya virus AF15561-infected mice^*a*^

Organ	Replicate no.	No. of reads			Median nt coverage
		Total	Host	Virus	
Serum	1	1,991,095	12,324,949	372,210	2,997.5
	2	4,914,494	10,770,576	3,914,465	31,977.5
	3	10,150,263	17,439,351	8,628,164	71,524
	4	7,835,883	7,601,111	6,761,112	55,618

SKM-CL	1	12,920,938	12,777,208	44,073	294
	2	10,884,345	10,491,084	1,538,267	12,460
	3	12,120,048	15,199,969	236,946	1,867.5
	4	12,243,525	7,495,822	309,850	1,863

SKM-IS	1	12,231,654	15728625	2,893,892	20,349
	2	15,379,613	14,818,574	1,665,210	14,151
	3	14,997,585	12,895,046	1,297,009	10,213
	4	8,801,363	1,078,426	1,739,616	13,525

Heart	1	13,286,951	1,391,885	225,684	1,182
	2	11,620,802	669,058	273,366	1,459.5
	3	18,709,794	1150507	223,552	1,388
	4	8,152,377	636,741	37,435	216

Kidney	1	13,810,118	12,107,848	277,261	1,553
	2	11,063,493	8,848,847	54,343	267
	3	16,439,432	11,317,154	297,215	2,017
	4	8,037,635	11,204,970	31,460	198

Spleen	1	6,362,763	8,714,336	9,324	75
	2	10,006,661	11,461,848	1,548	0
	3	8,877,156	13,106,104	730	0
	4	1,009,068	6,548,374	148	0

Liver	1	16,473,789	5,225,328	19,659	141
	2	15,456,139	6,370,167	13,516	88
	3	13,565,351	3,867,685	9,170	64
	4	1,652,843	530,545	484	0

a nt, nucleotide; SKM-CL, skeletal muscle from contralateral leg; SKM-IS, skeletal muscle from injection site.

**FIG 8:**
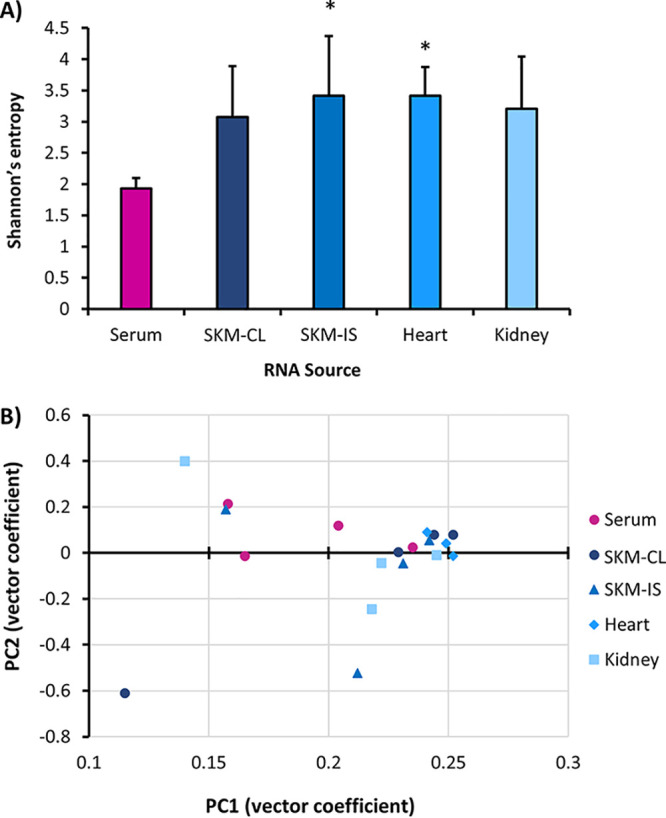
CHIKV D-RNA diversity and expression in a mouse model of infection. Four A129 mice were infected with CHIKV AF15561, and then serum and organs were collected 4 days post-infection. RNA was then extracted from serum and tissues and then sequenced on an Illumina NextSeq550. Defective RNAs (D-RNAs) were identified and quantified using ViReMa v1.5. From raw data, Shannon’s diversity index *H* (A), weighted for median nucleotide coverage, was calculated for individual data sets using a custom python script (Kruskal-Wallis with Dunnett’s *post hoc* test; *, *P* < 0.05). Additionally, D-RNA count data were analyzed using DESeq2, followed by principal-component analysis (B).

D-RNAs were identified in all tissues as well as serum, featuring the recombination events identified during *in vitro* studies. Consistent with our *in vitro* results, D-RNA diversity (as indicated by Shannon’s entropy) was higher in tissues (i.e., intracellularly) than in serum (virion RNAs) (Fig. 8A). Further, average serum diversity and average cell-derived P2 virion diversity indices were similar at 1.92 and 2.3, respectively. However, average tissue D-RNA diversity, ranging between 3.07 and 3.4, was lower than average cell-derived intracellular diversity at 6.8. While only heart muscle and skeletal muscle at the injection site had significantly higher diversity than serum (Kruskal-Wallis with Dunnett’s *post hoc* test, *P* < 0.05), these animals were not perfused prior to tissue collection, and thus, excess serum virus may have diluted the diversity of some tissue samples and subsequently affected standard deviation. Viremia ranged between 1.5 × 10^6^ and 1.2 × 10^8^ PFU/ml, and no correlation between diversity measures and viremia was observed (Fig. S8). Murine samples in general failed to cluster by organ (Fig. 8B), with the exception of heart samples, after PCA. Most tissue samples formed a single large cluster with one of the serum samples (from mouse 2), although serum samples in general showed no clustering pattern at all. Thus, although statistical significance was not achieved in all cases, diversity trends generally agree with those observed *in vitro*.

10.1128/mBio.00731-20.8 FIG S8 Relationship between organ-specific Shannon’s entropy (*H*) and viremia among mice infected with CHIKV. Four A129 mice were infected with CHIKV AF15561 in the left rear footpad. Animals were humanely euthanized, and serum and organs were collected 4 days post-infection. RNA was then extracted from serum and tissues and sequenced on an Illumina NextSeq550. Defective RNAs were identified and quantified using ViReMa v1.5. Viremia is plotted on the *x* axis, while Shannon’s entropy (*H*) is plotted on the *y* axis, with dotted trend lines in pink and various shades of blue and *R*^2^ values indicated next to trend lines. Download FIG S8, TIF file, 0.2 MB. Copyright © 2020 Langsjoen et al. 2020 Langsjoen et al. This content is distributed under the terms of the Creative Commons Attribution 4.0 International license.

**FIG 9:**
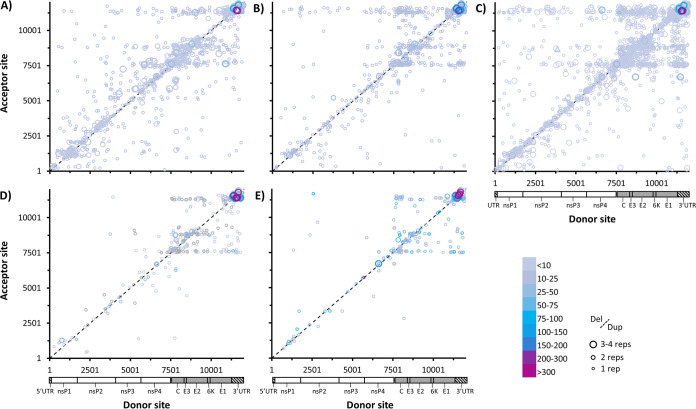
Recombination boundaries observed among CHIKV D-RNAs in a murine model of infection Four A129 mice were infected with CHIKV AF15561, and then serum and organs were collected 4 days post-infection. RNA was extracted from serum and tissues and then sequenced on an Illumina NextSeq550. D-RNAs were identified and quantified using ViReMa v1.5. CHIKV recombination junctions for D-RNAs with ≥1 count per every 2 × 10^6^ reads were then plotted for serum (A), contralateral skeletal muscle (B), injection site skeletal muscle (C), heart (D), and kidney (E). Circle size indicates number of replications, lines delineate deletion versus duplication-copyback events, and color indicates count/10^6^ mapped reads.

Also consistent with *in vitro* results, heat maps of recombination junctions reveal the same three acceptor sites observed *in vitro* across both serum and tissue samples, as well as a similar donor site specific to tissues rather than serum (Fig. 9). Interestingly, skeletal muscle, and especially kidney tissue, was particularly enriched for sgD-RNAs. 3′ UTR recombination events remained the most common type of D-RNA in all samples. This was also evident when the top deletion events were compared across different tissues, where kidney samples unsurprisingly most closely recapitulated *in vitro* data (Fig. S9 and S10). In addition to the 3′ boundary observed in the 3′ UTR, all mouse samples showed an additional 5′ boundary in the 3′ UTR. Not only did we observe similar trends in diversity measures between *in vitro* and *in vivo* studies, but we also observed similar trends in D-RNA population composition. Thus, we have demonstrated biologically relevant D-RNA phenomena that can be exploited for better understanding RNA virus replication, with potential applications in human health.

10.1128/mBio.00731-20.9 FIG S9 Top deletion events observed among CHIKV-infected mouse organs. Four A129 mice were infected with CHIKV AF15561 in the left rear footpad. Animals were humanely euthanized, and serum and organs were collected 4 days post-infection. RNA was extracted from serum and tissues and then sequenced on an Illumina NextSeq550. Defective RNAs were identified and quantified using ViReMa v1.5. The top 60 deletions for mouse 2 serum (A), contralateral leg muscle (B), injection site leg muscle (C), cardiac muscle (D), and mouse 1 kidney (E) (a low overall number of recombination events was observed in the M2 kidney sequencing reaction; therefore, to compare deletions, M1 kidney was used) are shown. (F) Hierarchical analysis of D-RNA expression and organ clustering for mouse 1. Download FIG S9, TIF file, 0.7 MB. Copyright © 2020 Langsjoen et al. 2020 Langsjoen et al. This content is distributed under the terms of the Creative Commons Attribution 4.0 International license.

10.1128/mBio.00731-20.10 FIG S10 Top insertion-duplication events observed among CHIKV-infected mouse organs. Four A129 mice were infected with CHIKV AF15561 in the left rear footpad, and then animals were humanely euthanized. Serum and organs were collected 4 days post-infection. RNA was then extracted from serum and tissues and sequenced on an Illumina NextSeq550 system. Defective RNAs were identified and quantified using ViReMa v1.5. The top 21 insertion/duplication events for mouse 2 serum (A), contralateral leg muscle (B), injection site leg muscle (C), cardiac muscle (D), and kidney (E) are shown. Download FIG S10, TIF file, 0.5 MB. Copyright © 2020 Langsjoen et al. 2020 Langsjoen et al. This content is distributed under the terms of the Creative Commons Attribution 4.0 International license.

## DISCUSSION

The goal of these studies was to determine how and whether intracellular D-RNA populations differ from virion populations, addressing whether D-RNAs can arise *de novo* without requiring packaging and subsequent accumulation through high-MOI passaging. By sequencing early-passage, low-MOI-derived stock, intracellular P1.5 virus, and subsequent P2 virion (passage 2 virus), we have shown that D-RNA diversity and composition are fundamentally different between intracellular and resulting P2 virion RNAs, both *in vitro* and *in vivo*, for 5 alphavirus strains from 4 distinct species and 2 distinct phylogenetic groups. As a result of these studies, we additionally observed a novel conserved subtype of alphavirus D-RNA originating from recombination events in the subgenomic region, specifically between the capsid-E3 region and the end of the E1-3′ UTR subgenomic region, which is heavily enriched intracellularly but does not appear to be commonly packaged. We call this new heterogeneous subpopulation subgenomic D-RNAs (sgD-RNAs).

Viruses, especially RNA viruses, are notorious for their ability to generate highly diverse populations, particularly in terms of point mutations that are generated during replication and may contribute to overall viral fitness within a host (62, 63). Although researchers tend to think of viral population dynamics in terms of P2 progeny viruses, there may be a role for intracellular RNA diversity that remains unexplored. Here, we show that D-RNA populations differ between intracellular and P2 virion factions in early viral passages. For all viruses tested, there were significantly more unique recombination events intracellularly than were packaged, indicating that only a portion of D-RNAs generated are packaged. Overall numbers of D-RNAs are additionally higher intracellularly, and thus, Shannon’s entropy index (*H*) is significantly higher in intracellular samples, indicating greater alpha diversity intracellularly. These trends held for *in vivo* studies, where D-RNA populations from specific tissues were more diverse than those from serum. Broadly, this indicates a packaging bottleneck between intracellular and P2 virion populations, which likewise means that production of D-RNAs occurs *de novo* without a strict requirement for transmitted template. These results also distinguish packaged D-RNAs and D-RNAs produced *de novo* as two separate populations. Packaged D-RNAs have been shown to play multiple roles during viral infection, which range from interfering with replication of wild-type parental virus RNA and promoting viral persistence in tissue culture to stimulating the host immune response *in vivo*. However, unpackaged D-RNAs have not been appreciated as a distinct population before now, and subsequently, the role of these D-RNAs remains unclear. These studies therefore provide a critical foundation for further investigation of the role of *de novo* D-RNA production during viral infection by establishing this important distinction between packaged and unpackaged D-RNAs.

There is some evidence that recombination events leading to D-RNA formation are largely, though not exclusively, polymerase driven (64–66) and are influenced by polymerase characteristics such as fidelity. For example, low-recombination variants of Senecavirus exhibited a high-fidelity polymerase phenotype (67), a low-fidelity SINV polymerase mutant showed increased rates of virion D-RNA production (34), and a high-fidelity poliovirus polymerase mutant exhibited decreased rates of recombination (68). A popular model for recombination events leading to D-RNA formation is through a copy choice mechanism, in which a polymerase retains the nascent RNA chain but switches templates during transcription (69, 70). In this model, it is generally thought that template switching occurs during antisense-strand synthesis, thus giving rise to a defective template prior to generation of daughter sense-strand RNA. This is a reasonable hypothesis for a few reasons, including the following: (i) antisense template is generally rare, affording few opportunities for recombination between discrete templates, and (ii) to produce D-RNAs to transmissible levels, presumably a defective template would be required to generate a minimum copy number to increase packaging efficiency. Kirkegaard and Baltimore were also able to demonstrate this experimentally using a wild-type poliovirus and a guanidine-resistant poliovirus mutant, which showed preferential template-switching activity consistent with that predicted for the generation of recombinant negative-sense template RNA (69).

However, the data presented here suggest that template switching may not occur exclusively during antisense template synthesis. First, that alphavirus D-RNA production likely occurs *de novo* implies that transmitted template is not a strict requirement for D-RNA production. In addition, differential D-RNA diversity between intracellular and P2 virion alphavirus RNA was due almost entirely to recombination events in the subgenomic region encoding the structural proteins. The packaging signals of alphaviruses are thought to be in the nsP1 (71, 72) and, in the case of Semliki Forest virus, nsP2 (3) genes in the nonstructural gene cassette; thus, the lack of packaging of these particular D-RNAs may be due to the fact that they are specifically on sgRNA transcripts and not genomic transcripts. This was confirmed by DRS, which showed that CHIKV sgDels were expressed primarily in sgRNA transcripts. Furthermore, since genomic and sgRNA transcripts are both transcribed from full-length negative-sense template RNA, we would reasonably expect to see the same deletions in both genomic and subgenomic reads in long-read sequencing data if template switching occurs during negative-sense strand synthesis; however, because almost no overlap in deletions was observed between genomic and subgenomic reads in either of our DRS data sets, we could not confirm the presence of a common defective template. Together, these results suggest that either (i) template switching can occur during either antisense- or sense-strand synthesis or (ii) sgD-RNAs are not generated through a copy choice mechanism. Clustering of recombination junctions into hot spots supports the former.

Similar to previous work, we also observed substantial recombination activity in the 3′ UTR of CHIKV. These CHIKV 3′ UTR recombination events mostly consisted of duplications, especially in intracellular populations. Duplications in the CHIKV 3′ UTR are well documented and are thought to influence CHIKV host adaptability, particularly in the insect vector (35, 37, 73). Filomatori and Bardossy and colleagues additionally found that these recombination events arise from a mixture of homologous- and nonhomologous-template switching (38), although the relative quantities of each varied depending on host type. In addition to these, we have also shown that CHIKV, MAYV, SINV, and AURV all readily produce sgD-RNAs featuring recombination events in the subgenomic region, especially subgenomic deletions (sgDels) during early, low-MOI passages, as well as in a murine model of CHIKV infection. All sgD-RNAs share similar boundaries between all viruses tested, particularly deletions with the first junction occurring between the capsid and E3 regions and the second occurring between E1-3′ UTR regions. Because these populations arise regularly not only after infection with different CHIKV stock viruses but also during infection with heterologous alphaviruses, with unpredictable overlap between those observed among intracellular and P2 virion RNAs, this suggests that they arise *de novo* and are consistently expressed at low levels intracellularly. The boundaries appear to be loosely conserved between the alphaviruses tested, which all either cause or are highly related to viruses that cause arthritogenic disease (57). Interestingly, 3′ UTR events were relatively enriched in P2 virion factions compared to sgDels and other subgenomic recombination events, potentially indicating a different mechanism through which these events occur.

Intriguingly, although this study focused on arthritogenic alphaviruses, the general sgD-RNA boundaries correspond to a well-known recombination event between Eastern equine encephalitis virus (EEEV) and SINV that gave rise to the Western equine encephalitis virus (WEEV) species (57, 74). Little is known about the particulars of this recombination event, such as whether it occurred in an insect or mammalian host, but determining the mechanism of expression of sgD-RNAs among alphaviruses may help delineate the circumstances that gave rise to WEEV. This is especially important, as CHIKV is now cocirculating with MAYV (75), Una virus (75), and Eastern and Venezuelan equine encephalitis viruses since its introduction to the Western hemisphere in 2013, presenting new opportunities for heterologous alphavirus recombination events that may gave rise to novel recombinant alphavirus species.

A common concern regarding research on D-RNAs is that they may simply be an artifact of tissue culture, given the contrived passaging conditions from which most D-RNAs have been identified and characterized. Whether D-RNAs arise during natural infection, and what roles D-RNAs may play, is therefore an important question. RSV D-RNAs have been recovered from patient samples and indeed have been correlated with an enhanced host response to infection (17, 76), while similar findings have been described for IAV (18). For alphaviruses, it was previously shown that salmonid alphavirus 3 forms many D-RNAs in wild salmon hosts (16), among which 6K deletion mutants are common (40). 6K deletion mutants have also been observed among VEEV RNA populations derived from sentinel hamster hosts (41). However, we have definitively shown that a pathogenic alphavirus, CHIKV, forms D-RNAs with substantial incapacitating deletions in a mammalian host. Importantly, many of the recombination events we observed *in vivo* were similar to ones observed *in vitro* during early passages, suggesting that expression of sgD-RNAs is a programmed phenomenon with potential biological functions, recapitulating observations made with IAV (20).

In all, the present study provides critical evidence of discrete types of D-RNAs, virion/transmissible and intracellular/*de novo-*produced, as well evidence for a novel type of alphavirus D-RNA, sgD-RNAs. Further study is required to ascertain the origin of alphavirus sgD-RNAs and the factors leading to formative recombination events. There are two general hypotheses that are not mutually exclusive: (i) that sgD-RNAs are an artifact of another replicative process, such as the binding of host and/or viral proteins to viral RNA, and (ii) that sgD-RNAs are a deliberate by-product of viral replication with specific cellular functions. Additionally, the function of alphavirus sgD-RNAs and other *de novo*-produced D-RNAs remains unknown, necessitating further investigation.

## MATERIALS AND METHODS

### Tissue culture.

Vero cells (American Type Culture Collection, Manassas, VA) were maintained in Dulbecco’s modified Eagle medium (DMEM; Gibco) containing 1× penicillin-streptomycin-amphotericin (PSA; Gibco, Waltham, MA) and 5% fetal bovine serum (FBS; HyClone, Logan, UT). Aedes albopictus mosquito (C7/10) cells, kindly provided by Scott Weaver, were maintained in DMEM containing 1× PSA, 10% FBS, 1× nonessential amino acids (Sigma, St. Louis, MO), 1× sodium pyruvate (Gibco), and 5 ml 1× tryptose phosphate buffer (Gibco).

### Viruses and infections.

CHIKV 181/clone 25 (47), CHIKV AF15561 (47), and MAYV IQU3056 (77) infectious clones were kindly provided by Scott Weaver. Low-passage-number AURV BeAr93375 (P2, C6/36) and SINV CGLT599 (P2, suckling mouse) isolates were provided by the World Reference Center for Emerging Viruses and Arboviruses (WRCEVA; Galveston, TX). Infectious clones were rescued as follows. First, plasmids were digested with appropriate endonuclease, and then digested plasmid was purified using phenol-chloroform-isoamyl alcohol. Then, 1 μg digested plasmid was added to a mMessage mMachine SP6 reaction mixture (Ambion, Austin, TX), followed by DNase digestion and RNA cleanup using an RNA Clean and Concentrator kit (Zymo, Irving, CA) following the manufacturer’s instructions. Cleaned RNA was electroporated into Vero cells using a Neon electroporation device following the manufacturer’s Vero cell protocol (Thermo Fisher Scientific, Waltham, MA). All viruses were amplified in C7/10 cells using an MOI of 0.1 to generate P1 stock virus; P0 (rescued clone or reconstituted stock from WRCEVA) and P1 stock titers were determined in Vero or BHK21 cells using plaque assays as previously described (78) and generally ranged between 10^6^ and 10^7^ PFU/ml from Vero cells and 10^7^ to 10^9^ PFU/ml from C7/10 cells.

Viral infections were performed as follows: virus was first diluted in serum- and antibiotic-free DMEM to a target MOI of 2 infectious units/cell. Culture medium was removed from cells, and virus medium was added for 1 h, rocking plates every 15 min. Cells were then washed 3 times with PBS, and normal culture medium was added.

### RNA extractions.

Intracellular RNA was collected 11 to 12 h post-infection as follows: supernatant was removed from target wells, followed by 3 washes with 2 ml PBS, and then 500 μl TRIzol (Invitrogen, Carlsbad, CA) was added directly to plate and incubated for 5 min prior to collection. Packaged RNA was collected 48 h post-infection as follows: supernatant was collected, clarified by centrifugation for 5 min at a relative centrifugal force (rcf) of 1,000, and incubated in 7% PEG 8000–NaCl overnight at 4°C. Precipitate was then pelleted by centrifugation at 3,200 × *g* for 30 min at 4°C, and supernatant was removed; the pellet was resuspended in 100 μl 10% FBS-DMEM and then incubated with 10 μg RNase A for 2 h at room temperature (to remove contaminating nonpackaged RNAs). TRIzol (500 μl) was then added to RNase-treated sample and incubated for 5 min. For both intracellular and packaged RNAs, RNA was then extracted using Phasemaker tubes (Invitrogen) according to the manufacturer’s instructions. Additionally, rRNA was removed from intracellular RNA samples using a Lexogen RiboCop kit according to manufacturer’s instructions (Lexogen Inc., Greenland, NH).

### Animal infections and RNA collections.

Type 1 interferon-deficient IFN-αR^−/−^ (A129) mice of different ages and genders were kindly provided by Slobodan Paessler. Mice were infected with 10^3^ PFU CHIKV AF15561 in 10 μl PBS in the left rear footpad and monitored for weight and signs of disease. Animals were euthanized 4 days post-infection by CO_2_ overdose followed by cardiac puncture, whereupon blood, skeletal muscle from the infected and contralateral legs, heart, kidney, liver, and spleen were collected; organs were placed in 300 μl RNAlater solution, while whole blood was centrifuged at an rcf of 3,380 for 5 min, and serum was collected and stored at −80°C until further use. Upon processing, tissue was removed from RNAlater solution, rinsed in sterile PBS, and placed in a 2-ml microcentrifuge tube with 300 μl Buffer RLT and a stainless steel ball bearing. Samples were heat inactivated at 60°C for 15 min, and then organs were homogenized using a TissueLyser II (Qiagen, Hilden, Germany) with shaking at 26 frequency 1/s for 5 min. RNA was then extracted from organs in Buffer RLT using RNeasy fibrous tissue minikits following the manufacturer’s instructions (Qiagen), while RNA was collected from serum using TRIzol according to the manufacturer’s instructions. Mouse studies were performed in an animal biosafety level 3 facility according to approved University of Texas Medical Branch (UTMB) Institutional Animal Care and Use Committee (IACUC) protocol number 1708051. The University of Texas Medical Branch complies with NIH policy on animal welfare, the Animal Welfare Act, and all other applicable federal, state, and local laws.

### Next-generation sequencing.

ClickSeq libraries were generated as previously described (42, 43). Oxford Nanopore Technologies’ direct RNA sequencing (DRS) libraries were constructed as follows: intracellular RNA was immediately ribo-depleted using Ribo-Zero kits (Illumina, San Diego, CA) upon RNA extraction and used within 24 h of ribodepletion (stored on ice). DRS kits (Oxford Nanopore Technologies, Oxford, UK) were purchased directly from ONT, and libraries were constructed using 250 to 300 ng of starting RNA according to the most recent version of the DRS protocols published by the manufacturer. The libraries were run on a MinION sequencer using R9.4.1 flow cells for 8 to 12 h each.

### Bioinformatics.

For AURV BeAr93375 and SINV CGLT599, corrected reference genomes were generated using Pilon based on publicly available sequences (79). For CHIKV 181/clone 25, CHIKV AF15561, and MAYV IQU3056 clones, the viral cDNA sequences provided by the Weaver lab were used for reference genomes. Reads were trimmed and quality filtered using the fastp tool’s default parameters (44), and then recombination events were identified and enumerated using our ViReMa (45) v1.5 integrated pipeline with the following inputs:[script location]/ViReMa.py [reference genome].txt [input file].fastq [output file designation].sam –Output_Dir [output folder] –p 4 –MaxIters 10 –MicroInDel_Length 5.

The resulting .txt files from ViReMa were used to calculate Shannon’s entropy (*H*), weighted by median nucleotide coverage, utilizing a previously described custom python script (43). Further, using DESeq2 (48), for each virus recombination events were compiled and normalized, and expression changes were evaluated based on the ViReMa recombination output file. From these results, hierarchical clustering was performed using Cluster 3.0 (49), filtering for a minimum of 2 replicates with expression values of >2; the resulting trees were visualized using TreeView (50). Additionally, normalized data from DESeq2 were used to perform principal-component analyses in SigmaPlot. ONT sequences were aligned to alphavirus genomes using minimap2 (52), and read alignments were extracted using a custom python script.

### Statistics.

All statistics were performed in SigmaPlot. For Shannon’s entropy and unique/total D-RNA comparisons, data were tested for normality using the Shapiro-Wilk test, followed by Student’s *t* test comparing intracellular and virion P2 samples. Animal data were tested for normality followed by one-way analysis of variance (ANOVA). Data formatted by DESeq2 were used for principal-component analyses performed in SigmaPlot.

### Data availability.

All raw data files for Illumina and default quality-filtered, base-called data for direct RNA nanopore sequencing data sets associated with this paper are available in the SRA NCBI archive under study number PRJNA613616.
